# Impact of a Canadian Pediatric Society Position Statement on Trampoline-related Injuries at IWK Health Centre, Halifax, Nova Scotia

**DOI:** 10.7759/cureus.2609

**Published:** 2018-05-10

**Authors:** Graham Wilson, Colleen Sameoto, Eleanor Fitzpatrick, Katrina F Hurley

**Affiliations:** 1 Division of Paediatric Emergency Medicine, IWK Health Centre; 2 Emergency Department, IWK Health Centre

**Keywords:** trampoline, injury prevention, advocacy, epidemiology, paediatric trauma

## Abstract

Introduction: In 2007, the Canadian Pediatric Society (CPS) advised against the recreational use of trampolines at home and reaffirmed that statement in 2013. We evaluated the impact of this position statement on trampoline-related injuries at the IWK Health Centre in Halifax, Nova Scotia.

Methods: We completed a retrospective analysis (2001-2015) using the IWK Health Centre‘s Canadian Hospitals Injury Reporting and Prevention Program (CHIRPP) database. The time frame was divided into a pre-statement, post-statement, and post-reaffirmed statement. We included data on captured emergency department visits for children aged 0-16 years with trampoline-related injuries. Demographics, location, and injury mechanism were compared using the chi-squared and z tests. The proportions of trampoline injuries for pre-, post-, and post-reaffirmed statements were compared via analysis of variance (ANOVA).

Results: Since the CPS statement, trampoline-related injuries significantly increased at the IWK Health Centre from 0.9% to 1.6% (p<0.001). Injuries increased in children under four years old and decreased in children 10-14 years (p=0.009). Recreational use at home (93%) remained the most common location of the incident (p<0.001). Fractures (n=277) and sprains/soft tissue injuries (n=232) to the ankle, head/neck, or elbow remained the most common injuries and did not significantly change post-statement or post-reaffirmed statement (p>0.05).

Conclusions: Despite the CPS statement, trampoline-related injuries remain an important source of injuries at the IWK Health Centre. The types of injury did not significantly change during this time frame.

## Introduction

While trampolines have been used recreationally since the 1950s [1], their use and resulting injuries have increased dramatically [2-6]. In the United States, trampoline injuries increased by 98% between 1990 and 1995 [3], with 731,247 trampoline-related injuries occurring between 2004 and 2014 [5-6]. Nationally, the Canadian Hospitals Injury Reporting and Prevention Program (CHIRPP) found an increase in the number of trampoline injuries from 459/10,000 in 1999 to 649/10,000 in 2005 [4]. Furthermore, trampoline-related injuries were more likely to result in a hospital admission when compared to other sports, with CHIRRP reporting a 56% increase in hospital admissions between 1990 and 2001 [4,7]. In one study, up to 61% of patients required surgery [8].

Commercial indoor trampoline parks are also becoming a source of trampoline-related injuries, with an estimated 345 parks being opened in the United States in 2014 [9]. Soft tissue injuries, fractures, and even death have been reported in such facilities [10-13]. The safety of these facilities has been questioned by the medical community [11-15].

While some feel that calling for an outright ban on trampoline use is too drastic, others believe that a ban on recreational trampoline use is the only solution [1-2,16-19]. In 2007 and again in 2013, the CPS published a position statement regarding trampoline safety [18]. It recommends against the purchase of trampolines for home use and suggested that health-care professionals warn parents of the dangers of recreational trampoline use at routine health visits. It also recommends that physicians advocate for legislation with respect to product warning labels. CPS has not yet made recommendations about the use of commercial indoor trampoline parks.

The purpose of this study was to evaluate the impact of this position statement and whether or not there has been a change in injury type, location, and/or numbers of trampoline injuries at the IWK Health Centre since the release of the 2007 CPS statement and 2013 reaffirmation [18].

## Materials and methods

The CHIRPP is an injury surveillance system operating in 11 pediatric emergency departments (EDs) and six general EDs in Canada. Information regarding the circumstances that led to the injury is documented using a questionnaire that is completed during the patient’s ED visit. The clinical data is added by an attending physician or staff, de-identified, and subsequently coded [20].

The injury data used in this study was obtained from the IWK Health Centre’s CHIRPP database. The IWK is the primary children’s health center in the Maritimes, providing care to women, children, and youth from Nova Scotia, New Brunswick, and Prince Edward Island. The population captured in this database includes any child presenting or referred to the IWK Health Centre ED.

Our dataset was extracted by one of the investigators (CS) using the following inclusion criteria: age less than 16 years; and, included the injury event description keywords: “trampoline,” “jump,” or “sault” or indicated trampolining as a factor. A case-by-case review of each record was performed by the same investigator to determine whether it fit the inclusion criteria.

The variables extracted for comparison included age, sex, year of injury, location of injury (e.g., home, school), the nature of injury (e.g., fracture, soft tissue injury), body part involved, and the mechanism of injury (e.g., falling off the trampoline). The total number of ED encounters per year for all injuries by age group was examined. Data were analyzed using SPSS 24.0 (IBM, Armonk, NY, US). Demographics, location, nature of injury, and injury mechanism were compared using the chi-square and z tests. The proportions of trampoline injuries (the total number of trampoline injuries over the total injuries at the IWK) were assessed pre-statement, post-statement, and post-reaffirmed statement via ANOVA and compared for differences with post hoc tests. Statistical significance was set at p=0.05 for all analyses.

We examined trampoline injuries captured between January 1, 2001. and December 31, 2015. This was then stratified into pre-statement (January 1, 2001 – July 31, 2007), post-statement (August 1, 2007 – December 31, 2012) and post-reaffirmed statement (January 1, 2013 – December 31, 2015). Study approval was received from the IWK Research Ethics Board.

## Results

**Figure 1:**
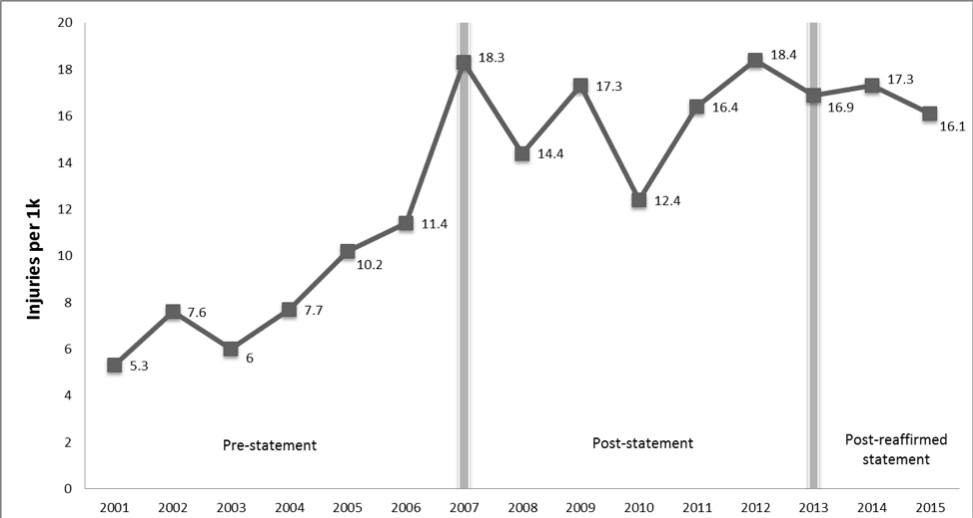
IWK trampoline-related ED visits, 2001-2015. Expressed as a proportion (per 1000) of all injury cases in the IWK CHIRPP database ED: emergency department; CHIRPP: Canadian Hospitals Injury Reporting and Prevention Program

Between 2001 and 2015, the IWK CHIRPP database provided injury data on 78,328 injuries with most injuries occurring in the 2002-2007 time block. Overall, there were 999 global trampoline-related injuries in the 14 years of data. The percentage of IWK trampoline-related ED visits significantly increased from 0.9% (n=363) pre-statement (PrS) to 1.6% (n=487) post-statement (PoS) and 1.6% (n=149) post-reaffirmed statement (PRS) (p<0.0001, Figure 1). Note that the time-frame for the PRS was two years compared to five years for PrS and PoS.

Females (n=540) experienced slightly more trampoline-related injuries than males (n=459) (Table 1). These injuries were most likely to take place at home (n=831) and in children between the ages of 10 and 14 (n=419). While most injuries occurred at home, the proportion of injuries at home decreased in PRS compared to PrS and PoS (92.9% PrS, 95.8% PoS, 84.1% PRS, p<0.05). The decrease in the proportion of trampoline-related injuries at home corresponded to an increase in injuries in recreational facilities. Fractures (n=451) were the most common injury and the ankle (n=213) was the most common body part involved. The most common mechanism of injury was landing incorrectly (n=284).

**Table 1:** Demographics and location of trampoline-related injuries from the IWK CHIRPP database (2001-2015) *Denotes a significant difference (p<0.05). CHIRPP: Canadian Hospitals Injury Reporting and Prevention Program

Variable	Time Frame		
	Pre-statement	Post-statement	Post-reaffirmed statement
	# cases (%)	# cases (%)	# cases (%)
Gender			
Male	161 (44.4)	225 (46.2)	73 (49.0)
Female	202 (55.6)	262 (53.8)	76 (51.0)
Age group			
<4	47 (12.9)*	90 (18.5)*	31 (20.8)*
5 to 9	122 (33.6)	194 (39.8)	60 (40.3)
10 to 14	176 (48.5)*	190 (39.0)*	53 (35.6)*
15 to 16	18 (5.0)	13 (2.7)	5 (3.43)
Location			
Home/other home	276 (92.9)*	439 (95.8)*	116 (84.1)*
School	11 (3.7)*	3 (0.7)*	3 (2.2)
Fitness facility	7 (2.4)	13 (2.8)	7 (5.1)
Sports/rec. area	2 (0.7)*	0 (0.0)*	10 (7.2)*
Campground	1 (0.3)	3 (0.7)	2 (1.4)

There were no statistically significant differences in injury by gender, nature of injury, or mechanism of injury when comparing PrS to PoS and PRS (p>0.05). Fractures (50.3% PrS, 47.9% PoS, 43.7% PRS) and sprains/soft tissue injuries (40.8% PrS, 38.5% PoS, 42.2% PRS) remained the most common trampoline-related injuries. The ankle (22.1% PrS, 20.0% PoS 22.4% PRS), head/neck (13.4% PrS, 14% PoS, 10.9% PRS), and elbow (11.2% PrS, 12.3% PoS, 11.6% PRS) were the most commonly injured body parts irrespective of timeframe. Forearm injuries decreased significantly from PrS to PRS (11.8% PrS, 5.1% PRS, p<0.05), while foot/toe injuries increased (8.1% PrS, 6.9% PoS, 13.8% PRS, p<0.05) (Table 2).

**Table 2:** Characteristics of trampoline-related injuries in the IWK CHIRPP database (2001-2015) *Denotes a significant difference (p<0.05).

Variable	Time Frame		
	Pre-statement	Post-statement	Post-reaffirmed statement
	# cases (%)	# cases (%)	# cases (%)
Nature of injury			
Superficial	12 (3.5)	24 (5.3)	8 (5.9)
Laceration	14 (4.0)	23 (5.0)	6 (4.5)
Fracture	174 (50.3)	218 (47.9)	59 (43.7)
Sprain/soft tissue	141 (40.8)	175 (38.5)	57 (42.2)
Concussion/minor head	5 (1.4)	15 (3.3)	5 (3.7)
Body part			
Head/neck	48 (13.4)	65 (14.0)	15 (10.9)
Chest/trunk	19 (5.3)	25 (5.4)	4 (2.9)
Shoulder/upper arm	8 (2.2)	20 (4.3)	6 (4.3)
Elbow	40 (11.2)	57 (12.3)	16 (11.6)
Forearm	42 (11.8)*	42 (9.1)	7 (5.1)*
Wrist	21 (5.9)	27 (5.8)	11 (8.0)
Hand/fingers	22 (6.2)	19 (4.1)	8 (5.8)
Hip/thigh	7 (2.0)	4 (0.9)	2 (1.4)
Knee	20 (5.6)	39 (8.4)	12 (8.7)
Lower leg	22 (6.2)	41 (8.8)	7 (5.1)
Ankle	79 (22.1)	93 (20.0)	31 (22.4)
Foot/toes	29 (8.1)	32 (6.9)*	19 (13.8)*
Mechanism of injury			
Fall OFF trampoline	64 (18.7)	64 (14.3)	22 (16.3)
Fall ON trampoline	94 (27.4)	119 (26.6)	27 (20.0)
Collision with person/object	66 (19.2)	100 (22.4)	30 (22.2)
Incorrect landing	100 (29.2)	140 (31.3)	44 (32.6)
Flips	19 (5.5)	24 (5.4)	12 (8.9)

In terms of age group (Table 1), there was a significant increase in the proportion of injuries occurring in children less than four years old (12.9% PrS, 18.5% PoS, 20.8% PRS, p=0.009) and a significant decrease in the proportion of injuries occurring in 10-14 year olds (48.5% PrS, 39.0% PoS, 35.6% PRS, p=0.009). Of the known locations where the trampoline injury occurred (Table 1), there was no significant change between the pre-statement and post-statement timeframes in term of home injuries (92.9% PrS vs. 95.8% PoS); however, there was a significant decrease in the proportion of injuries occurring at home during the post-reaffirmed statement timeframe (84.1%, p<0.05). There were also a lower proportion of injuries in school (3.7% PrS vs. 0.7% PoS, p<0.05) in the post-statement period. After the reaffirmed position statement, there was a significant increase in injuries occurring at a sports or recreational facility (0.7% PrS vs. 0.0% PoS vs. 7.2% PRS, p<0.05).

Eighty-five percent of all trampoline injuries happened on the trampoline itself. Falling on the trampoline (24%), incorrectly landing (28%), and colliding with another person (20%) remained the most common mechanisms of injury and did not significantly change following the CPS position statement (Table 2).

## Discussion

Since the 2007 position statement by the CPS (reaffirmed in 2013), trampoline-related injuries seen in the ED of the IWK Health Centre have significantly increased. Despite the majority of injuries occurring in children 5-14 years of age, as previously reported [1-2,4,8,21-25], our study found a significant increase in the number of injuries that occurred in children under four years old. This is particularly concerning since one study has shown that the smallest child is 14 times more likely to be injured when multiple jumpers are on a trampoline, as was the case in 23.2%-77% of studies [1-2,8,24,26].

When CHIRPP data was nationally reported in 2006, trampoline injuries were increasing (420 per 100,000 CHIRPP cases in 1999 compared to 799 per 100,000 CHIRPP cases in 2006) [4]. Despite the CPS position statement in 2007, we demonstrated a continued increase in trampoline-related injuries in our catchment. Reasons for this continued increase are unclear. Have we failed in translating the knowledge to health-care providers who are expected to advocate to prevent these injuries? Or have health-care providers failed to impress upon parents the importance of preventing these injuries?

One may relate this increase to a lack of the adult supervision of children using trampolines. Studies have found that adult supervision is present in 27%-77.4% of injuries [16,21,23-24], raising questions as to whether supervision is really the issue since it is unlikely to prevent all injuries [1,16,27]. The use of safety measures, such as netting, can lull some parents into a false sense of security [25,28]. One study found that parents believed that trampoline injuries occurred only from falling off the trampoline, making the presence of a net enough to eliminate the risk of injury [25]. Alexander et al. found that pads and netting around the trampoline did not reduce the frequency of injury [26]. In our study, only 15% of injuries were associated with falling off the trampoline. Some speculate that adult supervision may help prevent injuries in which the trampoline is used in "imaginative" ways, including jumping from the roof, playing under the trampoline while others are jumping, and bringing objects onto the trampoline [1].

An interesting finding in our data was that although recreational use at home remained the most common location of the incident, more injuries are occurring in sports facilities, potentially in locations like trampoline parks. A trampoline park opened in the Halifax area in November 2015, a month before the end of our capture period. Unlike recreational formats, commercial trampoline parks consist of multiple adjoining trampolines in an open concept, with additional trampolines being built on angles and onto walls, permitting greater vertical and lateral movements [14]. This format permits the combination of trampolines with other sports, such as dodgeball and basketball [26], and encourages multiple jumpers, a situation associated with increased risk of injury [1-2,4,6,8,16,21,23-25,26]. Position statements by the CPS, as well as other education and public health initiatives, have focused on trampoline use at home, encouraging precautions such as adequate supervision, no somersaults, shock-absorbing pads and enclosure netting [10,14]. Unfortunately, these standards do not extend to commercial trampoline parks [7,13-14].

There are limitations to our study. The search string that we used may not have caught all injury data related to trampoline use; however, it was performed according to the national CHIRPP standard [4]. Our dataset was derived from ED visits to a single facility. Due to age limitations at the IWK Health Centre, this study does not capture injuries suffered by older teenagers and underrepresents injuries that occur outside the Halifax Regional Municipality. Moreover, it cannot account for trampoline injuries that occurred in children who did not present to the ED. At the time of data collection, expressed consent was required to be included in the IWK CHIRRP database and, as a result, major trauma is not captured in our database. The IWK is a major referral center for specialized management and, thus, fractures may be overrepresented. We cannot comment on specific patient characteristics, such as socioeconomic status and parental education, since the CHIRRP database focuses on injury data.

No causal inferences can be made about the CPS statements and trampoline use, as we did not verify whether the patient or caregivers were exposed to or aware of the CPS policy statement, nor did we evaluate whether physicians in the community knew about the statement and advocated for safe trampoline practices in their interactions with families.

## Conclusions

The increasing rate of trampoline-related injuries is cause for concern. Extremity fractures comprise a large number of trampoline-related injuries. While padding, netting, and compliance with manufacturer guidelines may improve trampoline safety, these measures are not consistently used and, when in place, cannot prevent all injuries. Since the reaffirmed CPS position statement (2013), home-based trampoline injuries at our institution decreased, while the overall number of trampoline-related injuries increased. Targeted risk reduction approaches need further study, as does the impact of recreational trampoline facilities. Further study as to the dissemination and uptake of national guidelines and practice statements is needed to determine whether they effectively reach the target audiences: practitioners and the public.

## References

[1] Woodward GA, Furnival R, Schunk JE (1992). Trampolines revisited: a review of 114 pediatric recreational trampoline injuries. Pediatrics.

[2] Furnival RA, Street KA, Schunk JE (1999). Too many pediatric trampoline injuries. Pediatrics.

[3] (2016). US Consumer Product Safety Commission. https://cpsc.gov/recall-products/trampolines.

[4] (2016). Injuries Associated with Backyard Trampolines: Canadian Hospitals Injury Reporting and Prevention Program (CHIRPP) database, 1999-2003 (cumulative to February 2006), update 2004-2006, 1749 cases. http://www.phac-aspc.gc.ca/injury-bles/chirpp/injrep-rapbles/pdf/trampolines-eng.pdf.

[5] Briskin S, LaBotz M (2012). Trampoline safety in childhood and adolescence. Council on sports medicine and fitness. Pediatrics.

[6] Kasmire KE, Rogers SC, Sturm JJ (2016). Trampoline park and home trampoline injuries. Pediatrics.

[7] McFaull SR (2007). Injuries associated with backyard trampoline use, Canadian Hospitals Injury Reporting and Prevention Program (CHIRPP), 1999-2003. Abstract presented at the 84th Canadian Paediatric Society Annual Conference, June 26-30, 2007, Montreal, Quebec. Paediatr Child Health.

[8] Sandler G, Nguyen L, Lam L, Manglick MP, Soundappan SS, Holland AJ (2011). Trampoline trauma in children: is it preventable?. Pediatr Emerg Care.

[9] Sarris Sarris (2016). Jump! Spin! Fly!. Internet]. Jump! Spin! Fly! [updated April.

[10] Mulligan C, Adams S, Brown J (2016). Paediatric injury from indoor trampoline centres. Inj Prev.

[11] Cardozo G (2016). Families call for urgent safety review of Central Coast Flip out indoor trampoline centres. Injury Prevention.

[12] (2016). Trampoline park injuries spark safety probe and hospital investigation. The Sydney Morning Herald. 2014.

[13] Stern R. Maureen Kerley Pushes for Trampoline Park Regulations Following 2012 (2018). 'They are very dangerous': trampoline park death highlights calls for regulation. Available from http://www.phoenixnewtimes.com/news/maureen-kerley-pushes-for-trampoline-park-regulations-following-2012-death-of-son-at-phoenixs-skypark-6663641.

[14] (2016). Trampoline safety. https://www.canada.ca/en/health-canada/services/healthy-living/your-health/products/trampoline-safety.html.

[15] Williams Williams (2016). Trampoline parks great exercise as long as you don't get hurt. 3.

[16] Smith GA, Shields BJ (1998). Trampoline-related injuries to children. Arch Pediatr Adolesc Med.

[17] Brown PG, Lee M (2000). Trampoline injuries of the cervical spine. Pediatr Neurosurg.

[18] Purcell L, Philpott J (2016). Trampoline use in homes and playgrounds. Paediatr Child Health.

[19] Hammer A, Schwartzbach AL, Paulev PE (1982). Some risk factors in trampolining illustrated by six serious injuries. Br J Sports Med.

[20] Crain J, McFaull S, Thompson W, Skinner R, Do MT, Fréchette M, Mukhi S (2016). The Canadian Hospitals Injury Reporting and Prevention Program: a dynamic and innovative injury surveillance system - HPCDP: Volume 36-6, June 2016. Health Promot Chronic Dis Prev.

[21] Klimek PM, Juen D, Stranzinger E, Wolf R, Slongo T (2013). Trampoline related injuries in children: risk factors and radiographic findings. World J Pediatr.

[22] Black GB, Amadeo R (2003). Orthopedic injuries associated with backyard trampoline use in children. Can J Surg.

[23] Leonard H, Joffe AR (2009). Children presenting to a Canadian hospital with trampoline-related cervical spine injuries. Paediatr Child Health.

[24] Hurson C, Browne K, Callender O (2007). Pediatric trampoline injuries. J Pediatr Orthop.

[25] Wootton M, Harris D (2009). Trampolining injuries presenting to a children's emergency department. Emerg Med J.

[26] Alexander K, Eager D, Scarrott C, Sushinsky G (2010). Effectiveness of pads and enclosures as safety interventions on consumer trampolines. Injury Prevention.

[27] (2016). Positions Statement: Trampolines and Trampoline Safety. https://www.aaos.org/uploadedFiles/PreProduction/About/Opinion_Statements/position/1135%20-%20Trampolines%20and%20Trampoline%20Safety.pdf.

[28] Linakis JG, Mello MJ, Machan J, Amanullah S, Palmisciano LM (2007). Emergency department visits for pediatric trampoline-related injuries: an update. Acad Emerg Med.

